# An Unusual Presentation of Charcot Arthropathy Caused by Syringomyelia Mimicking a Soft Tissue Tumor

**DOI:** 10.1155/2014/760861

**Published:** 2014-07-13

**Authors:** Cuneyd Gunay, Ebru Atalar, Baybars Ataoglu

**Affiliations:** ^1^Department of Orthopaedic Surgery and Traumatology, Ankara Numune Training and Research Hospital, Ülkü Mah. Talatpasa Bulvarı No. 5, Altındağ, 06100 Ankara, Turkey; ^2^Department of Physical Therapy and Rehabilitation, Ankara Atatürk Chest Diseases and Chest Surgery Training and Research Hospital, 06280 Ankara, Turkey; ^3^Department of Orthopedic Surgery and Traumatology, Gazi University School of Medicine, 06560 Ankara, Turkey

## Abstract

Charcot arthropathy is a chronic, degenerative condition and is associated with decreased sensorial innervation. Numerous causes of this arthropathy have been described. Here we report a case of neuropathic arthropathy secondary to syringomyelia which was misdiagnosed as a soft tissue tumor and treated surgically and additionally with radiotherapy at another institution. The patient had clinical and radiological signs of syringomyelia, associated with a limited range of motion, swelling, and pain in the affected joint. Neuropathic arthropathy, although less common, should be considered in cases of unexplained joint swelling, pain, and limited range of motion of the affected joint.

## 1. Introduction

Neuropathic arthropathy, also known as Charcot joint, is a destructive situation resulting from decreasing or loss of proprioception, pain, and temperature sensation [1]. Many diseases which lead to neural damage, such as diabetes mellitus, tabes dorsalis, and syringomyelia, can be the cause of neuropathic arthropathy [2]. Occasionally, it is associated with leprosy, amyloidosis, peripheral nerve injury, myelomeningocele, spinal cord injury, familial dysautonomia, and congenital insensitivity to pain [3]. Neuropathic arthropathy is seen in 25% of all cases with syringomyelia, and 80% of those occur in the upper extremity. Shoulder joint involvement has been reported in 6% of patients with neuropathic arthropathy [4–7]. The aim of this study was to present a patient with Charcot arthropathy caused by syringomyelia, who had been initially misdiagnosed at another institution and treated surgically and with radiotherapy for a soft tissue tumor of the shoulder joint. In this report, the clinical findings, previous treatment, initial diagnosis, and work-up options are discussed.

## 2. Case Report

A 44-year-old female patient was admitted to our clinic in 2012 with left shoulder pain which had been present for 1 year. Past medical history revealed that she had been examined at a state hospital in her hometown 9 months previously with complaints of shoulder pain. After magnetic resonance imaging (MRI) examination, a preliminary diagnosis was made of malignant mass in the shoulder joint. A tru-cut biopsy was performed, after which she underwent surgery for aggressive fibromatosis. Despite radiotherapy treatment for 6 months after surgery, her condition gradually worsened. During follow-up, an MRI study was carried out for suspected recurrences and, as a result, she was referred to our hospital with a diagnosis of recurrence of soft tissue tumor.

**Figure 1:**
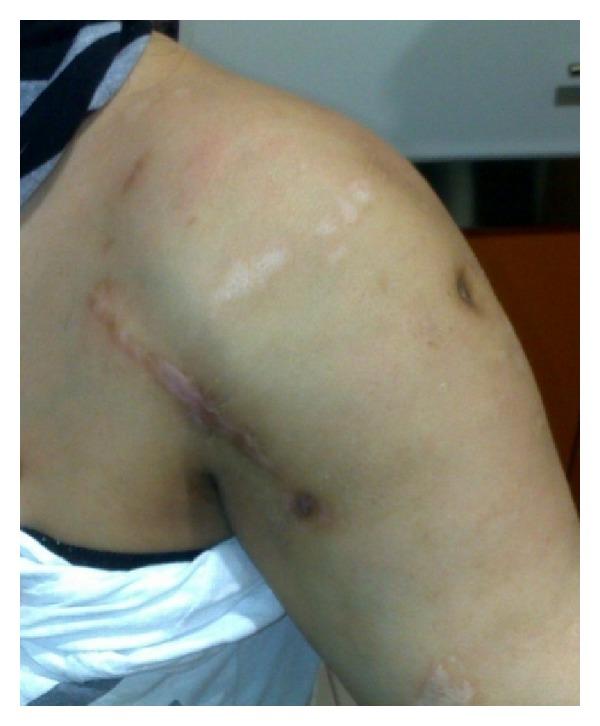
Clinical photograph of a 44-year-old female patient. The deltopectoral scar incision, which was performed surgically due to misdiagnosis of soft tissue tumor, can be seen.

**Figure 2:**
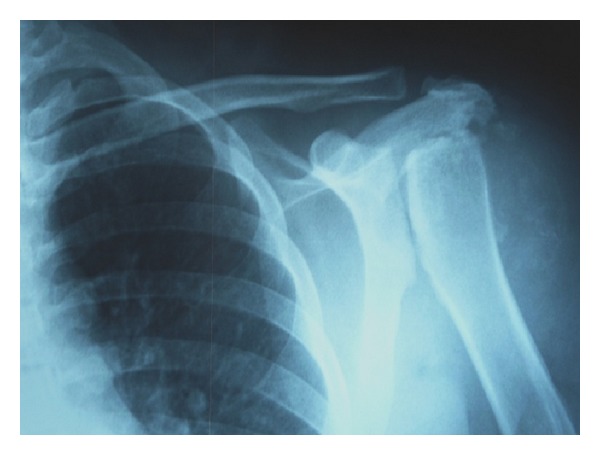
Radiography of the left shoulder revealed severe destructive changes with loss of bone and joint architecture, consistent with a Charcot joint.

**Figure 3:**
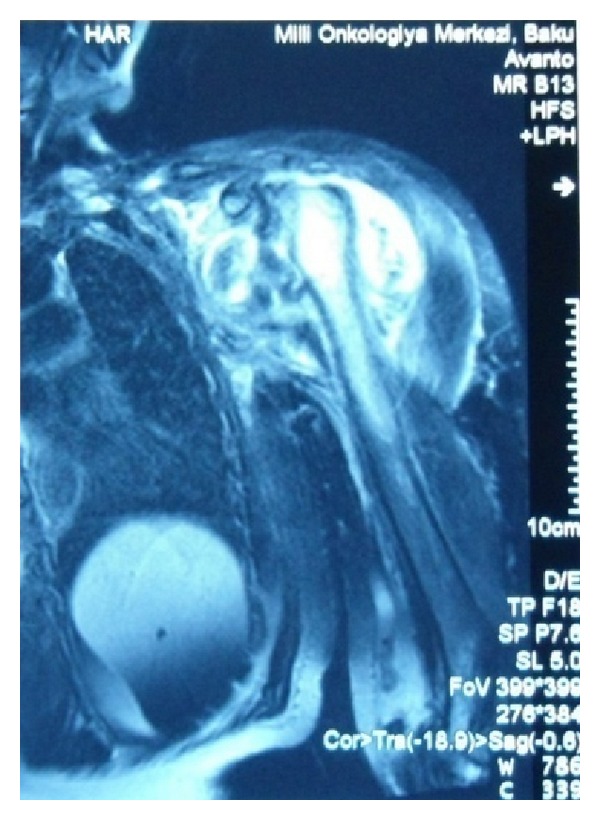
Axial MRI of the left shoulder revealed destruction of the humeral head, synovial hypertrophy, a large amount of joint effusion, and an irregular mass formation.

**Figure 4:**
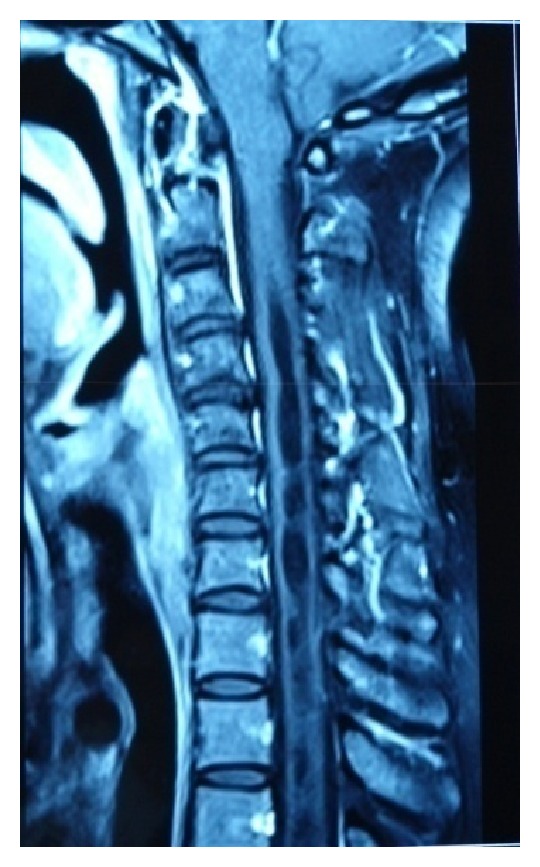
T2-weighted sagittal magnetic resonance imaging (MRI) of the cervical spine revealed a syrinx throughout the cervical cord, with extension into the upper thoracic region with mild diffuse volume loss.

On physical examination, a deltopectoral incision scar, swelling, and superficial skin lesions, which were probably secondary to radiotherapy, were observed (Figure 1). Neurological examination showed hypoesthesia and loss of temperature sensation in the left shoulder. Range of motion for passive movements was significantly higher than for active movements. Passive shoulder abduction was 45°, forward elevation was 40°, external rotation was 15°, and internal rotation was at the gluteal region. Muscle strength around the left shoulder joint was determined as 2-3/5. All the hematological investigations were within normal limits, on the basis of which syphilis and diabetes were ruled out. Radiographs of the shoulder joint showed destruction of the humeral head, osteolysis in the glenoid cavity, and areas of calcification on the soft tissue (Figure 2). Shoulder MRI revealed marked fluid collection, intra-articular synovial hypertrophy, destruction of the humeral head, and a large lobular irregular mass formation (Figure 3). The preliminary diagnosis at our clinic was neuropathic arthropathy of the shoulder joint. Cervical MRI was obtained to investigate the etiology. MRI revealed a spinal syrinx from cervical-3 (C3) to thoracic-1 (T1) vertebral levels (Figure 4). MRI of the thoracic spine did not demonstrate further extension of the syrinx, and the brain showed no intra- or extra-axial lesions or Arnold-Chiari malformation. To rule out other diagnostic possibilities, an open biopsy was performed under general anesthesia, and focal chronic inflammation, synovial hypertrophy, chronic synovitis, and necrotic bone were found. No mycobacterial or other organisms grew on cultures.

After evaluation of all the findings, the patient was diagnosed with neuropathic shoulder arthropathy and was referred to the physical therapy and rehabilitation department for conservative treatment. Nonsteroidal anti-inflammatory drugs were started for swelling and pain of the shoulder. In addition, the patient was managed conservatively for prevention of joint trauma with physical therapy and a protective orthosis was applied. At the two-year follow-up, the shoulder pain had subsided and mobility had improved. On physical examination, passive forward flexion and abduction were 90°, external rotation was 25°, and internal rotation was at the infrascapular region. Muscle strength around the shoulder was 3-4/5. At the time of writing this report, the patient was doing well on this conservative mode of treatment, performing daily activities and fully capable of independent self-care.

## 3. Discussion

The diagnosis of neuropathic arthropathy is often delayed. In this case study, a patient with Charcot arthropathy of the shoulder joint caused by syringomyelia was initially misdiagnosed and treated for a soft tissue tumor. Syringomyelia is a chronic and slowly progressive disease of the spinal cord in which there is a fluid-containing cavity (syrinx) inside the spinal cord. It can be congenital or can occur due to trauma, vascular problems, tumor, degeneration, or infection [8]. Neuropathic arthropathy is a process of chronic degenerative arthropathy which is associated with sensorial loss of the involved joint [1]. There are two theories describing the pathogenesis of neuropathic arthropathy. The neurotraumatic theory, first described in 1967, involves repetitive trauma sustained by an insensitive joint [1]. The other theory, reported by Allman et al. in 1988, is the neurovascular theory, describing active bone resorption by osteoclasts, secondary to sympathetic dysfunction [2].

There are many causes of this arthropathy and up to 25% of cases of neuropathic arthropathy are caused by syringomyelia [6]. A neuropathic joint disease may develop early or late in the course of syringomyelia. Neuropathic arthropathy of the shoulder generally progresses slowly, although rapid progression may occur over months or even weeks [9]. The symptoms of neuropathic shoulder arthropathy may mask the symptoms of syringomyelia. Due to neurological symptoms occurring at a late stage, patients initially consult an orthopaedic surgeon related to their shoulder pain [4]. For patients, admitted to orthopaedic clinics suffering from pain, swelling, and loss of range of movement of the shoulder, a differential diagnosis of neuropathic arthropathy of the shoulder should be considered, and a thorough neurological examination should be made. If a diagnosis of neuropathic shoulder arthropathy is considered likely after the evaluation of X-rays and MRI, a cervical MRI should be taken to rule out syringomyelia, which is the most common underlying disease.

Diabetic patients tend to have involvement of the joints of the foot and ankle, whereas larger joints such as the knee are commonly affected in patients with syphilis [3]. Shoulders and elbows are mostly involved, and the hand, wrist, or cervical spine has been reported less frequently [4, 10]. Effusion, soft-tissue swelling, narrowing of the joint space with subchondral sclerosis, and osteophytes are possible early radiographic findings of a neuropathic arthropathy and thus a differentiation between primary osteoarthrosis and neuropathic arthropathy is often difficult, especially in the early stages. Shoulder MRI showing periarticular fluid collection and mass formation resembles chronic septic arthritis or sarcoma, as in the current case. The differential diagnoses to be considered are septic arthritis, soft tissue sarcoma, idiopathic osteolysis, nephropathy, synovial chondromatosis, Winchester syndrome, or Gorham disease [3, 5]. Clinically, neuropathic joints are characterized by pain and deformation together with a limited range of motion [10, 11]. The patient presented here had clinical and radiological signs of syringomyelia, associated with a limited range of motion, swelling, and pain in the affected joint.

Some authors have reported unsatisfactory results with arthrodesis, resurfacing operations, and hemiarthroplasty, which have been applied for the treatment of neuropathic shoulder arthropathy [12, 13]. In this case, the deformed shoulder joint was treated conservatively and this resulted in an acceptable level of function with tolerable pain. The primary goal of conservative treatment is to reduce further articular damage by the prevention of repetitive trauma. Physical therapy with the use of braces can be considered to be probably the best solution for long-term management of these cases.

In conclusion, Charcot's shoulder is a rare disorder described in fewer than 60 patients in world literature, and correct diagnosis is possible by careful physical and neurological examination and pathological evaluation when needed. Once a neuropathic joint has been diagnosed, its etiology should be ruled out with aseptic joint aspiration to look for infection or tumor, and an MRI to evaluate for syringomyelia if the etiology remains in doubt. In order not to miss the diagnosis, the orthopedic surgeon should be aware of the characteristic clinical and radiological signs of this rare condition.
